# Reliability and validity of a computer game-based tool of upper extremity assessment for object manipulation tasks in children with cerebral palsy

**DOI:** 10.1177/20556683211014023

**Published:** 2021-06-02

**Authors:** Anuprita Kanitkar, Sanjay T Parmar, Tony J Szturm, Gayle Restall, Gina Rempel, Nilashri Naik, Neha Gaonkar, Nariman Sepehri, Bhavana Ankolekar

**Affiliations:** 1 Department of Applied Health Sciences, University of Manitoba, Winnipeg, Canada; 2 SDM College of Physiotherapy, Shri Dharamshala Manjunatheshwara University, Dharwad, India; 3 College of Rehabilitation Sciences, University of Manitoba, Winnipeg, Canada; 4 Pediatrics & Child Health, University of Manitoba, Winnipeg, Canada; 5 Department of Physiotherapy, Ushas School for Exceptional Children, Hubli, India; 6 SDM College of Physiotherapy, Dharwad, India; 7 Faculty of Engineering, University of Manitoba, Winnipeg, MB, Canada

**Keywords:** Cerebral palsy, children, manual dexterity, computer game-based assessment, telemonitoring, telerehabilitation

## Abstract

**Introduction:**

A computer game-based upper extremity (CUE) assessment tool is developed to quantify manual dexterity of children with Cerebral Palsy (CP). The purpose of this study was to determine test-retest reliability of the CUE performance measures (success rate, movement onset time, movement error, and movement variation) and convergent validity with the Peabody Developmental Motor Scale version 2 (PDMS-2) and the Quality of Upper Extremity Skills Test (QUEST).

**Methods:**

Thirty-five children with CP aged four to ten years were tested on two occasions two weeks apart.

**Results:**

CUE performance measures of five chosen object manipulation tasks exhibited high to moderate intra-class correlation coefficient (ICC) values. There was no significant difference in the CUE performance measures between test periods. With few exceptions, there was no significant correlation between the CUE performance measures and the PDMS-2 or the QUEST test scores.

**Conclusions:**

The high to moderate ICC values and lack of systematic errors indicate that the CUE assessment tool has the ability to repeatedly record reliable performance measures of different object manipulation tasks. The lack of a correlation between the CUE and the PDMS-2 or QUEST scores indicates that performance measures of these assessment tools represent distinct attributes of manual dexterity.

## Background

Cerebral Palsy (CP) is one of the most common motor neurodevelopmental disorders which affects five of every thousand live births in India and three of every thousand live births in North America.^1,2^ Children with CP often have deficits in the performance of fine and gross motor skills of the upper extremities.^3^ Manual dexterity—the ability to perform functional tasks with the hands—is an important predictor of success in daily activities, participation in school, and social activities.^4^ Children with motor impairments of the upper extremity (UE) benefit from intensive, task-specific therapy programs such as constraint-induced movement therapy^5^ and hand arm bimanual intensive therapy.^6^ However, it is difficult to engage children with CP in therapy for long periods and sustain motivation for the intense repetitive task practices. Thus, there is a need for innovative therapeutic approaches that engage children to participate in long-term exercise programs and provide opportunities to improve neurodevelopmental outcomes.

An emerging approach to engage children in therapy is to incorporate computer game aided exercise methods in which a range of interactive challenges help children to participate in repetitive motor activities. Studies have provided evidence that well designed interactive computer games can improve players’ motor skills and participation in play as well as other daily activities.^5^ Ideally, these types of effective therapy programs should be made available in patients’ homes, including those in rural communities. With appropriate and reliable telemonitoring tools, remote telerehabilitation programs can be monitored by clinicians. For this purpose, an innovative computer game rehabilitation (CGR) platform has been developed.^7^ The CGR includes an embedded game-based assessment subsystem that automatically records the player’s performance and computes the outcome measures of object manipulation tasks. It is vital to obtain objective outcome measures and track changes over time to guide and progress individualized home programs. Therefore, exercise programs delivered in the home—or within rural communities—can be monitored and managed regularly by clinicians or by rural outreach programs.

The computer game based upper extremity (CUE) assessment tool uses a miniature, wireless inertial based (IB) computer mouse, which links physical movements with interactive computer games. When the IB mouse is attached to a test object, the natural motion of the object is then used to control the motion of the computer cursor or a game paddle. The CUE assessment is guided by standardized computer game-based activities and can be used with many different objects with a broad range of physical properties (e.g. weight, size, shape,) and functional demands. The CUE software is configurable, which helps to increase the difficulty level by adjusting a) the target and paddle size (for movement precision), b) movement speed of game objects, and c) mouse sensitivity (increase/decrease movement amplitude). The CUE software automatically logs the client’s movement response to each game event and computes various performance-based metrics such as Success Rate, Movement Onset Time, Movement Error, and Movement Variation. These performance metrics are averaged over a number of game events.^8,9^ For example, in one minute of gameplay, there will be 30 game events and corresponding movement responses. Thus, the CUE assessment tool can provide objective, performance-based outcome measures for many different goal-oriented object manipulation tasks for children with impairments of the upper extremity.

The purpose of this study is to examine the test-retest reliability and convergent validity of the CUE assessment tool. This is an essential initial step before the tool can be used routinely in clinical practice and within home telerehabilitation applications. The first objective of this study is to examine the test-retest reliability of the CUE assessment tool specifically for several goal-directed object manipulation tasks. It was hypothesized that CUE performance measures exhibit high test-retest reliability. The second objective was to assess the convergent validity of the CUE with the Peabody Developmental Motor Scale version 2 (PDMS-2) and the Quality of Upper Extremity Skills Test (QUEST). The PDMS-2 is a validated and commonly used assessment tool of hand use in young children that includes tasks such as throwing and catching a ball, stringing beads, and stacking blocks.^10–12^ Another commonly used and validated pediatric assessment of hand use is the QUEST.^13^ This assessment evaluates the range of motion and the type of grasp used in the handling and manipulation of three objects: a cube, a crayon, and a pellet.^11^ The CUE assessment tool focuses on different types and characteristics of hand use than do the PDMS-2 or the QUEST. Therefore, it is speculated that a significant but low correlation (Pearson r-value less than 0.5) will be observed.

## Methods

Ethical approval for the study was obtained from the Institutional Ethical Committee at both sites; S.D.M. College of Medical Sciences and Hospital Ethics Committee in Dharwad, India and the Health Research Ethics Board of the University of Manitoba, Canada. The institutional Research Board protocol number Is H2016:391 (HS20193) Parents of all study participants provided informed consent for the study. The Clinical trial registration number is. Clinicaltrials.gov NCT02728375; https://clinicaltrials.gov/ct2/show/NCT02728375 (Archived by Web Site at http://www.webcitation.org/6qDjvszvh)

## Participants

Thirty-five consecutive children diagnosed with CP attending the physiotherapy clinic who met the inclusion criteria volunteered to participate in the present study (Parental consent and child ascent was provided. Children were identified by pediatricians and recruited from the Outpatient Physiotherapy Department at SDM medical college and Hospital, Dharwad and Usha’s school for exceptional children, Hubli between April 2016 and March 2017. The following inclusion criteria was used:

1. Gross Motor Function Classification Scale (GMFCS) level I–III^14^
2. Manual Ability Classification System (MACS) level I–III^15^
3. Modified Ashworth scale was used to determine the level of spasticity in biceps brachialis, pronators and finger flexors, from grade 1 to 1+^16^
4. The pediatric version of the Mini-Mental State Exam for children (MMC) score above 17^17^
5. Children with right hand dominance

Exclusion criteria used:

1. Visual or auditory impairment or with a communication disorder that would prevent a child from being able to follow instructions or comprehend the computer games
2. Recent orthopedic impairment or botulinum toxin injection to the upper extremity within the last six months
3. History of convulsion disorder within the last six months

## Tests and instrumentation

The children attended two assessment sessions, Two weeks apart. The tests were performed on the same day of the week. The first assessment session included the following assessments in order: (a) Grasp and Visual-motor integration (VMI) tasks of the PDMS-2 [10], (b) QUEST grasp tests [11], and (c) the CUE assessment. The second CUE assessment was conducted by the same assessor after a two-week time period had elapsed.

**Figure 1.**
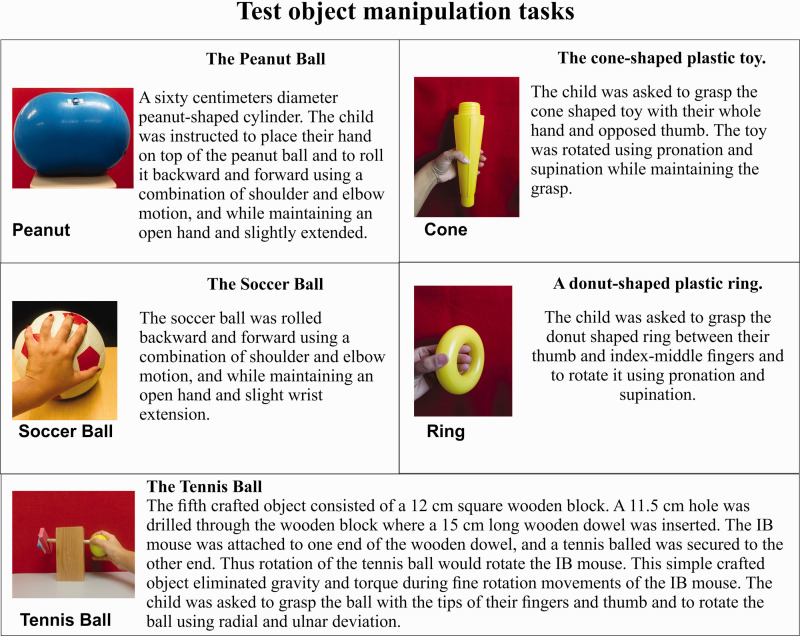
Describes the five test object manipulation tasks; peanut ball, soccer ball, tethered tennis ball, cone, and plastic ring.

The CUE assessment tool uses a wireless inertial-based mouse (Scoop™ Pointer Remote Model: RXR1000-0302E, Hillcrest Labs) which links physical movements with interactive computer games. The IB mouse contains a multi-axis gyroscope and firmware that detects instantaneous angular position which causes the movement of a computer cursor or a game paddle. Five test objects with different physical properties and anatomical demands were instrumented with the IB mouse. Figure 1 illustrates and describes the five test object manipulation tasks; peanut ball, soccer ball, tethered tennis ball, cone, and plastic ring. A custom game-based computer application was used to guide the object manipulation tasks of the CUE assessment the software presents a target object appearing at random locations at the top of the display screen. The object moves to the bottom of the display screen within two seconds of appearing and then disappears. One game event is defined as the time between the appearance and disappearance of a target. The children use the mouse to move the game paddle and catch the moving target objects.

## Protocol

**Figure 2.**
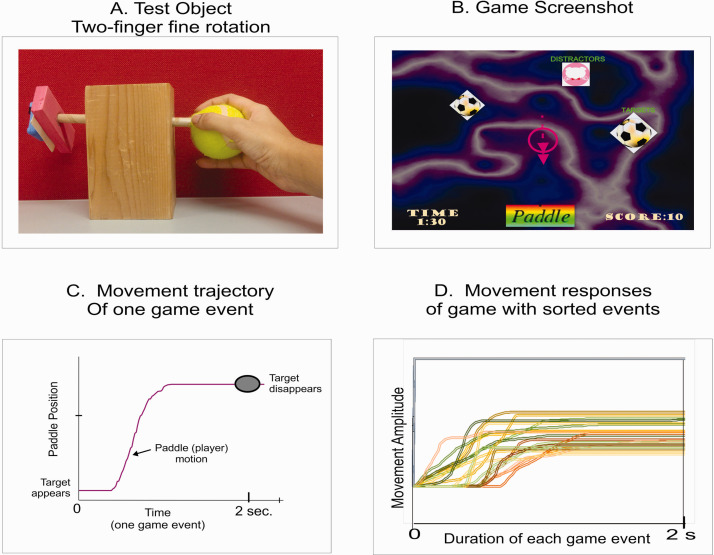
(a) Presents a screenshot of the CUE game and a plot of the trajectory of a typical paddle movement (motion of the test object) of one game event; (b) presents the trajectory of a single game movement response; (c) presents a single game trajectory of one game event; (d) presents overlay plots of all game movement responses for each direction obtained from one game session.

The children were allowed to play the CUE assessment game with a standard optical mouse until they became familiar with the game activities. During the assessment, participants were comfortably seated at a table with adjustable height. Each of the five test objects was placed on the table at a comfortable reaching distance within arm’s length of the participants. The order in which the different objects were assessed was kept constant between the first and second assessment session and the same clinician conducted both assessments. The CUE assessment tool is computer controlled and the coordinate data of the movements are automatically logged by the computer (see Figure 2). The performance measures were quantified off line by an analysis software using Mat Lab programs.

## Data processing and outcome measures

The grasping and visual-motor integration tasks of the PDMS-2 are scored with three levels: a score of zero if the child is unable to perform the task, a score of one of the task is partially performed, or a score of two if the task is completed. There are no time constraints given so the child may take any amount of time they require to complete or perform the given task. Only the raw scores were considered valid instead of the composite scores as the validity of the composite scores of PDMS-2 have only been evaluated in children five years of age and younger. The raw scores are recommended for use in older children with impairments.^10^ The QUEST compares the type of hand grasps used by the child to pick up three objects: a pencil or crayon, a cube, and a pellet. Scoring is based on how typical—or close to typical—the child’s grasp of each object is measured on a five-point grading scale.^13^

Figure 2 presents a screen shot of the CUE assessment game and individual game movement trajectories by direction for one CUE game session. The duration of each game event lasted 2 seconds and the game was played for 60 s, thus, 30 contextual movement responses are recorded for analysis, one-half in each direction. For a detailed description of the game movement indexing and segmentation see.^18^ Figure 2(d) presents overlay plots of individual game movement trajectories by direction for one CUE game session of 60 s. The following outcome measures of structure and function were derived from the recorded game movement responses of each test object manipulation task:

1. *Success rate* (SR) is the percentage of the total number of Target Objects that were caught in one game session.
2. *Average movement onset time* (MOT), which is the time from target appearance to the start of the game paddle movement. Values for MOT time are determined for each individual game movement response, and then the average is computed over the group of game movement responses for each direction.
3. *Movement error:* When a target is missed, the magnitude of the error (distance between paddle and target position) is. The average value for all misses is then computed as the Movement Error. Units are % of screen width.
4. *Movement variation:* The individual movement traces for each direction in one game session are averaged and the standard deviation computed for each sampled data point. The standard deviation value for all sampled data points is summed to obtain the total variance. This is reported as the movement variation. Units are % of screen width.

## Statistical analysis

The sample size for this study was computed using Table 1 from Zou et al.^19^ Thirty-five participants were required for an intra-class correlation coefficient (ICC) value of 0.8, assurance of 70%, and class-interval half-width of 0.15.

**Table 1.** Demographic and clinical characteristics of participants with cerebral palsy.

Participant	Age in years mean (SD)	Gender	GMFCS level	MACS level	Mean (SD)MMSE score	PDMS-2 Grasp score	PDMS-2 VMI score	QUEST Grasp Score
*N* = 35	7.5 (2.32)	F = 12, M = 23	I = 9II = 13III = 13	I = 12II = 14III = 9	31.7 (4.4)	42.02 (11.1)	109.8 (34.2)	63.7 (22.4)

Normality of the data was checked using the Shapiro–Wilk’s Normality test. Results determined the data was normally distributed. Relative reliability was assessed using a two-way random model intra-class correlation coefficient (ICC). The ICC scores were considered to be high when equal to or greater than 0.70, moderate between 0.5 and 0.69, and low when less than 0.50.^20^ Absolute reliability was analyzed using Minimal Detectable Change (MDC).^21–23^ Systematic errors between the test periods were evaluated using a paired t-test. Convergent validity of the CUE outcome measures with the PDMS-2 and QUEST scores was analyzed using Pearson’s correlation coefficient (R).^23^ The SPSS software for Windows, version 20.0 (SPSS Inc. Chicago) was used for all statistical analysis procedures.

## Results

Table 1 presents the demographic and clinical data. The mean age was 7.5 years and a standard deviation of 2.3 years. Twice as many boys were recruited as compared to girls. Three-quarters of the children were at GMFMC level II or III.

Tables 2 [Table table3-20556683211014023][Table table4-20556683211014023][Table table5-20556683211014023]to 6 present the results of the test-retest reliability analysis of the four CUE outcome measures for the five object manipulation tasks. The following summarizes the test-retest reliability results for success rate:

**Table 2.** Peanut ball manipulation task.

Variables	ICC	Mean and SD (Test 1)	Mean and SD (Test 2)	MDC	P value
MOT (RF)	0.70	0.92 (0.20)	0.93 (0.19)	0.27	0.001
MOT (RB)	0.24	0.91 (0.21)	0.86 (0.21)	0.42	0.23
SR (RF)	0.5	42.59 (20.99)	42.6 (21.48)	34.64	0.03
SR (RB)	0.45	45.05 (20.96)	43.11 (19.18)	36.37	0.06
ME (RF)	0.66	0.23 (0.10)	0.22 (0.1)	0.14	0.002
ME (RB)	0.72	0.24 (0.13)	0.22 (0.11)	0.16	0.001
MV (RF)	0.75	0.28 (0.1)	0.28 (0.1)	0.12	0.0001
MV (RB)	0.74	0.27 (0.1)	0.28 (0.09)	0.12	0.0001

Results of statistical analysis, ICC scores, group means and standard deviation (SD), minimal detectable change (MDC), and p-value of paired t-test comparing Tests 1 and 2 for Success Rate (SR), Movement Onset Time (MOT), Movement Error (ME) and Movement variation (MV). Data is presented during roll forwards (RF) as well as roll backwards (RB).

**Table 3. table3-20556683211014023:** Soccer ball manipulation task.

Variables	ICC	Mean and SD	Mean and SD	MDC	P value
MOT (RF)	0.72	0.97 (0.22)	0.96 (0.23)	0.28	0.0001
MOT (RB)	0.78	0.88 (0.15)	0.91 (0.15)	0.16	0.0001
SR (RF)	0.21	45.5 (18.19)	50.66 (14.6)	37.85	0.27
SR (RB)	0.6	44.6 (17.7)	45.59 (14.1)	26.48	0.01
ME (RF)	0.52	0.2 (0.1)	0.23 (0.1)	0.20	0.027
ME (RB)	0.76	0.25 (0.13)	0.25 (0.12)	0.14	0.0001
MV (RF)	0.78	0.30 (0.1)	0.32 (0.09)	0.11	0.0001
MV (RB)	0.83	0.305 (0.10)	0.32 (0.09)	0.1	0.0001

Results of statistical analysis, ICC scores, group means and standard deviation (SD), Minimal Detectable Change (MDC), and p-value of paired t-test comparing Test 1 and Test 2 for Success Rate (SR), Movement Onset Time, (MOT) Movement Error, (ME) and Movement variation(MV). Data is presented during roll forwards (RF) as well as roll backwards (RB).

**Table 4. table4-20556683211014023:** Ring manipulation task.

Variables	ICC	Mean and SD	Mean and SD	MDC	p-Value
MOT (CP)	0.239	0.82 (0.16)	0.86 (0.17)	0.32	0.233
MOT (ES)	0.46	0.89 (0.15)	0.92 (0.15)	0.26	0.052
SR (CP)	0.283	51.89 (25.23)	43.71 (24.37)	49.85	0.188
SR (ES)	0.60	51.62 (19.2)	52.64 (19.97)	29.1	0.012
ME (CP)	0.70	0.23 (0.14)	0.227 (0.14)	0.18	0.001
ME (ES)	0.727	0.20 (0.11)	0.20 (0.12)	0.13	0.0001
MV (CP)	0.64	0.28 (0.09)	0.29 (0.07)	0.12	0.004
MV (ES)	0.717	0.281 (0.09)	0.3 (0.09)	0.15	0.001

Results of statistical analysis, ICC scores, group means and standard deviation (SD), Minimal Detectable Change (MDC), and p-value of paired t-test comparing Test 1 and Test 2 for Success

Rate (SR), Movement Onset Time (MOT), Movement Error (ME) and Movement variation (MV). Data is presented during concentric pronation (CP) and eccentric supination (ES).

**Table 5. table5-20556683211014023:** Cone manipulation task.

Variables	ICC	Mean and SD	Mean and SD	MDC	P value
MOT (CP)	0.71	0.89 (0.18)	0.87 (0.17)	0.23	0.001
MOT (ES)	0.33	1.35 (2.51)	0.91 (0.12)	4.8	0.0534
SR (CP)	0.50	41.55 (19.70)	41.49 (21.74)	32.40	0.032
SR (ES)	0.1	29.66 (18.79)	34.47 (21.55)	41.60	0.0391
ME (CP)	0.50	0.28 (0.15)	0.27 (0.15)	0.24	0.043
ME (ES)	0.34	0.83 (0.7)	0.27 (0.12)	5.12	0.0136
MV (CP)	0.78	0.27 (0.09)	0.28 (0.08)	0.09	0.0001
MV (ES)	0.54	0.28 (0.08)	0.27 (0.08)	0.13	0.055

Results of statistical analysis, ICC scores, group means and standard deviation (SD), Minimal Detectable Change (MDC), and p-value of paired t-test comparing Test 1 and Test 2 for Success Rate (SR), Movement Onset Time, (MOT) Movement Error, (ME) and Movement variation(MV). Data is presented during concentric pronation (CP) and eccentric supination (ES).

**Table 6.** Tennis ball manipulation task.

Variables	ICC	Mean and SD	Mean and SD	MDC	P-value
MOT (CP)	0.60	0.89 (0.24)	0.91 (0.21)	0.36	0.004
MOT (ES)	0.60	0.86 (0.19)	0.84 (0.13)	0.28	0.89
SR (CP)	0.39	53.26 (24.10)	59.51 (22.7)	44.11	0.098
SR (ES)	0.41	49.72 (20.1)	57.34 (21.12)	40.81	0.82
ME (CP)	0.60	0.22 (0.12)	0.21 (0.13)	0.19	0.011
ME (ES)	0.34	0.22 (0.13)	0.20 (0.14)	0.25	0.0138
MV (CP)	0.75	0.26 (0.1)	0.28 (0.1)	0.12	0.0001
MV (ES)	0.65	0.27 (0.09)	0.28 (0.09)	0.13	0.003

Results of statistical analysis, ICC scores, group means and standard deviation (SD), Minimal Detectable Change (MDC), and p-value of paired t-test comparing Test 1 and Test 2 for Success Rate (SR), Movement Onset Time, (MOT) Movement Error, (ME) and Movement variation(MV). Data is presented during concentric pronation (CP) and eccentric supination (ES).

1. Test-retest reliability was high (ICC > 0.7, p ≤ 0.05) for manipulation of the peanut ball for both directions, roll backward direction of the soccer ball, concentric supination of cone and ring, and concentric ulnar deviation of the tethered tennis ball.
2. Test-retest reliability was moderate (ICC = 0.5–0.7, p ≤ 0.05) for roll forward direction of the soccer ball, eccentric pronation of cone and ring, and eccentric radial deviation of the tethered tennis ball.

The following summarizes the findings for the movement onset time outcome measure:

1. Test-retest reliability was moderate (ICC = 0.5–0.7, p ≤ 0.05) for manipulation of the peanut ball, soccer ball, cone, ring, and tethered ball in both directions except for the roll backward direction of the soccer ball, for which the test-retest reliability was high (ICC > 0.7, p ≤ 0.05).

The following summarizes the test-retest reliability results for movement error:

1. Test-retest reliability was high (ICC > 0.7, p ≤ 0.05) for the roll backward direction of the soccer ball, eccentric pronation of cone and ring, and eccentric radial deviation of the tethered tennis ball.
2. Test-retest reliability was moderate (ICC = 0.5 to 0.7, p ≤ 0.05) for manipulation of the peanut ball for both directions, roll forward direction of the soccer ball, concentric supination of cone and ring, and concentric ulnar deviation of the tethered tennis ball.

The following summarizes the test-retest reliability results for movement variation:

1. Test-retest reliability was high (ICC > 0.7, p ≤ 0.05) for the roll backward direction of a soccer ball and concentric ulnar deviation of a tethered tennis ball.
2. Test-retest reliability was moderate (ICC = 0.5–0.7, p ≤ 0.05) for the peanut ball, roll forward direction of the soccer ball, the cone manipulation for both directions, concentric supination of ring manipulation task, and eccentric radial deviation of a tethered tennis ball.

Results of the paired student’s t-tests showed no significant difference in any of the CUE assessment outcome measures between the first and second tests. This was the case for all object-manipulation tasks.

For the majority of cases, the minimal detectable change as a percentage of the group means (%MDC) was less than 30% for average movement onset time of the peanut ball and soccer ball roll backward task cone concentric supination, and ring eccentric supination. The %MDC was more than 60% for (a) success rate of cone eccentric pronation, ring eccentric pronation, tethered tennis ball eccentric radial deviation and (b) movement error of peanut ball in both directions, soccer ball roll forward task, cone concentric supination, ring manipulation in both directions, and tethered tennis ball concentric ulnar deviation.

### Convergent validity

Results of the correlation analysis between the CUE outcome measures and the PDMS-2 and QUEST test scores are presented in Tables 7 [Table table8-20556683211014023][Table table9-20556683211014023][Table table10-20556683211014023]to 11. Ninety of the 100 cases had r-values less than 0.3, and 48 of the 100 cases had r-values less than 02. There are also a number of correlations with an inverse relationship i.e. as performance of the CUE assessment improves the PDMS-2 and QUEST tests scores decline, or vice versa. Six of the 20 cases Between Success Rate and PDMS-2/QUEST test scores had negative r-values. Fifteen of the 20 cases Between Movement Error and PDMS-2/QUEST test scores had positive r-value. The majority of cases were not found to be significant. Sixteen of 20 possible comparisons between the CUE outcome measures and the PDMS-2 Grasp test score were not significant. Nineteen of 20 possible comparisons between the CUE outcome measures and the PDMS-2 VMI test score were not significant. Nineteen of 20 possible comparisons between the CUE outcome measures and the QUEST test score were not significant.

**Table 7.** Results of the correlation analysis between the CUE outcome measures of the peanut ball manipulation task and PDMS-2/QUEST scores.

	PDMS2_GRASP		PDMS2_VMI		QUEST_GRASPS		QUEST_TOTAL	
	Pearson correlation	Sig. (one-tailed)	Pearson correlation	Sig. (one-tailed)	Pearson correlation	Sig. (one-tailed)	Pearson correlation	Sig. (one-tailed)
AMO	–0.457*	0.01	–0.19	0.19	–0.01	0.47	0.10	0.32
SR	0.411*	0.02	0.28	0.09	–0.04	0.42	0.20	0.18
ME	–0.006	0.49	–0.19	0.19	0.26	0.12	0.17	0.22
MV	0.261	0.12	0.003	0.50	0.30	0.08	0.24	0.13

Presented are the Pearson correlation r-values and significance levels (p-values).*p value is significant.

**Table 8. table8-20556683211014023:** Results of the correlation analysis between the CUE outcome measures of the soccer ball manipulation task and PDMS-2/QUEST scores.

	PDMS2_GRASP		PDMS2_VMI		QUEST_GRASPS		QUEST_TOTAL	
	Pearson correlation	Sig. (one-tailed)	Pearson correlation	Sig. (one-tailed)	Pearson correlation	Sig. (one-tailed)	Pearson correlation	Sig. (one-tailed)
AMO	0.03	0.44	–0.24	0.13	–0.11	0.30	0.01	0.47
SR	0.08	0.35	0.22	0.15	–0.12	0.29	0.002	0.49
ME	0.22	0.15	0.11	0.30	0.37*	0.04	0.16	0.23
MV	0.009	0.48	–0.16	0.23	–0.25	0.12	–0.18	0.19

Presented are the Pearson correlation r-values and significance levels (p-values).*p value is significant.

**Table 9. table9-20556683211014023:** Results of the correlation analysis between the CUE outcome measures of the cone manipulation task and PDMS-2/QUEST scores.

	PDMS2_GRASP		PDMS2_VMI		QUEST_GRASPS		QUEST_TOTAL	
	Pearson correlation	Sig. (one-tailed)	Pearson correlation	Sig. (one-tailed)	Pearson correlation	Sig. (one-tailed)	Pearson correlation	Sig. (one-tailed)
AMO	–0.34	0.05	0.02	0.46	–0.04	0.42	–0.17	0.22
SR	0.28	0.09	0.23	0.15	–0.18	0.21	0.03	0.43
ME	–0.14	0.25	–0.24	0.13	0.46*	0.01	0.36*	0.04
MV	–0.27	0.11	–0.33	0.06	0.12	0.29	0.13	0.27

Presented are the Pearson correlation r-values and significance levels (p-values).*p value is significant.

**Table 10. table10-20556683211014023:** Results of the correlation analysis between the CUE outcome measures of the ring manipulation task and PDMS-2/QUEST scores.

	PDMS2_GRASP		PDMS2_VMI		QUEST_GRASPS		QUEST_TOTAL	
	Pearson correlation	Sig. (one-tailed)	Pearson correlation	Sig. (one-tailed)	Pearson correlation	Sig. (one-tailed)	Pearson correlation	Sig. (one-tailed)
AMO	0.09	0.33	0.12	0.30	0.01	0.48	–0.06	0.38
SR	0.19	0.19	0.06	0.39	–0.42*	0.02	–0.23	0.14
ME	0.07	0.37	–0.07	0.37	0.46*	0.01	0.31	0.08
MV	–0.01	0.46	–0.28	0.10	0.07	0.36	0.09	0.34

Presented are the Pearson correlation r-values and significance levels (p-values).*p value is significant.

**Table 11.** Results of the correlation analysis between the CUE outcome measures of the tennis ball manipulation task and PDMS-2/QUEST scores.

	PDMS2_GRASP		PDMS2_VMI		QUEST_GRASPS		QUEST_TOTAL	
	Pearson correlation	Sig. (one-tailed)	Pearson correlation	Sig. (one-tailed)	Pearson correlation	Sig. (one-tailed)	Pearson correlation	Sig. (one-tailed)
AMO	0.04	0.42	–0.09	0.34	0.05	0.41	0.03	0.43
SR	–0.23	0.15	0.02	0.47	–0.52**	0.006	–0.55**	0.004
ME	0.30	0.08	0.04	0.42	0.49**	0.009	0.48*	0.02
MV	0.01	0.48	–0.05	0.41	0.15	0.24	0.2	0.18

Presented are the Pearson correlation r-values and significance levels (p-values).*p value is significant.

## Discussion

With few exceptions, the CUE assessment tool showed high to moderate test-retest reliability for all of the object manipulation tasks, and there was no significant difference in mean scores between Tests 1 and 2. Minimal detectible change values were notably large for most of the object manipulation tasks.

Several gaming systems have been used as rehabilitation tools for assessment and treatment.^24–27^ These gaming systems detect and quantify arm segment motions or finger motions. The corresponding segment motion signals are used to interact with virtual avatars/objects, or to control a game paddle for play. However, these systems, do not involve object handling and manipulation. The present CUE assessment tool targets object handling and manipulation to extend the utility beyond gross reaching or finger movements since the ability to perform manual dexterity skills with the hands is very much an integral part of everyday life, in particular, precise, timely accurate movements. Importantly, the CUE assessment tool allows one to test many objects with varied physical and functional demands. Hundreds of objects of different mass, shape, and size, surface friction can be changed to function exactly as a computer mouse simply by attaching the miniature motion mouse. The five manipulation tasks tested in the present study were chosen to span a broad range of functional properties and anatomical requirements. Rolling the peanut and soccer ball forward and backward were similar and involved a combination of shoulder and elbow flexion and extension movements, while keeping the hand in contact with the surface and the fingers extended. As the soccer ball was manipulated forward and backward, the children were also required to prevent the ball from rolling sideways, left and right. Rolling the ball in one direction while minimizing its motion in other directions required more control than rolling of the peanut ball, i.e. a cylinder moves only about a single axis. Irrespective of the increased degrees of freedom, ICC values were similar for the peanut ball and soccer ball rolling task. The tethered tennis ball rotation task also could only rotate about a single fixed axis, and mass and torque were eliminated. Fine rotation of the tennis ball was produced by radio-ulnar deviation while maintaining the wrist in extension. Outcome measures of this task also had high to moderate ICC values.

The interactive CUE software provides several different standardized computer-guided activities, time constraints, and analysis procedures to objectively quantify various motor performance metrics. Four performance-based outcome measures were quantified in the present study: the success rate of each game event, movement onset time, movement error, and movement variation. These performance metrics represent different features of the signals recorded and different aspects of the processing involved, i.e. goal attainment, information processing and motor planning time, spatial aspects of movement precision, and movement consistency as measured over multiple responses. In the present study, the duration of each game event lasted 2 s and the game was played for 60 s, thus, 30 contextual movement responses are recorded for analysis, 15 in one direction, and 15 in the other. In this case, Success Rate is based on 30 game movement responses and Movement Onset, Movement Error, As well as Movement Variation are the averages of five movement responses in each direction.

When evaluating the effectiveness of treatment programs, performance measures having high MDC values of greater than 20% would require a greater amount of change from pre- to post-intervention for them to be significant.^26^ Minimal detectible change is dependent on both the level of correlation between the first and second tests (ICC value) and on the variation among subjects, i.e. group standard deviation. In the present study, the standard deviation for most CUE variables was quite high and ranged from 20 to 60% as a percentage of the group average. In an attempt to reduce the variation among subjects and to see if %MDC decreased, a subgroup analysis was performed on the cone and ring performance measures for children in MACS level I (*n* = 12) and MACS level II (*n* = 14). The results for the ring and cone object manipulation task showed that for all outcome measures the standard deviation and the %MDC for MACS level I and II subgroups either increased or stayed the same as compared to the whole group analysis. For example, standard deviation as a percentage of the group average for Success Rate was 34% for a cone for MACS I. It was unexpected that the standard deviation in performance measures for the manipulation tasks among the children in MACS level I did not decrease from the variance observed for the whole group MACS levels I to III. By definition, MACS level I includes children who can handle objects with minimal difficulty, however, for the object manipulation tasks tested in the present study goal attainment (Success Rate) was low with a range of 29–59 with a relatively large variation among the children in MACS level I. These findings were also true for the children in the MACS level II subgroup. Similar testing on typically developing children between four and ten years old (*n* = 40) using the same object manipulation tasks and gameplay configurations have been conducted by our group. The analysis revealed significant differences between children with CP and typically developing age matched controls in all performance measures. For example, the success rate for the typically developing controls was 88% ± 14%). Tasks, where performance is initially poor, i.e. success rate in the present study ranged from 29 to 59%, may show significant and clinically important changes with training. For example, a modest increase in success rate from 40 to 60% would represent a percentage increase of 50% relative to the pre-intervention value. This would exceed the %MDC values that were observed in the present study.

Results show that there is no linear relationship between CUE outcome measures and the PDMS-2 or QUEST test scores. Infact, for a number of cases the relationship was inverse i.e. improvements in CUE outcome measures was associated with declines in performance of the PDMS-2 and QUEST. Judging from the r-values presented in Tables 7 [Table table8-20556683211014023][Table table9-20556683211014023][Table table10-20556683211014023]to 11 indicates that the strength of association between these measures is low to very low. Taken together this would signify that the CUE assessment tool assesses different attributes of object manipulation skills than PDMS-2 and QUEST, or that the skills required to manipulate the test objects of the present study are different than the ones tested in the PDMS-2 or QUEST. With few exceptions there were no significant correlations found between the CUE assessment outcome measures and the PDMS-2 or QUEST scores. This would signify that the CUE assessment tool assesses different attributes of object manipulation skills than QUEST and PDMS-2, or that the skills required to manipulate the test objects of the present study are different than the ones tested in the QUEST or PDMS-2. During the PDMS-2 assessment, children are given credit for partial completion of the respective items but the criteria provided contain limited information or descriptors of the quality of the performance expected. For example, performance metrics, such as the movement onset time as well as the number and magnitude of movement errors, are not determined by the scoring method of the PDMS-2. In the QUEST assessment, participants are not required to manipulate the object. The game activities of the CUE assessment tool have specific goals and time constraints which can be adjusted. The speed of the game objects, paddle size, and movement amplitude can be varied depending on the task requirements and level of impairment. In addition, the tasks are repeated multiple times, and averages are obtained across the total data.

### Limitations

One main limitation of the CUE is that it requires an IB computer mouse, a computer, and basic knowledge of computer operation. Several fine motor skills cannot be assessed using the CUE assessment tool, such as buttoning and unbuttoning, tying shoelaces, and cutting food. Another limitation is that the inertia-based mouse detects angular motion, therefore, it is not possible to practice tasks that require only linear motion.

Another limitation is statistical uncertainty related to the assumptions of randomization and identically distributed samples. Particular one limitation of the present study is the selection bias of recruiting consecutive children, and recruitment from only one pediatric physiotherapy center versus a random selection of the population. However there is no reason to believe that the children were connected in any way (i.e. independent samples). The distribution of subjects, by age and impairment levels would have an impact on the results and would-be a limitation of this study. Pacifically, analysis of assessment tools using subjects that are homogeneous tend to have poorer ICC than those that utilize more heterogeneous distributions of subjects. Future studies should use a more homogeneous distribution of ages and impairment levels to minimize subject variation.

### Future implications

The CUE assessment tool is designed to be used as a part of a computer game based rehabilitation platform for fine motor function impairments in children with CP. Such integrated platforms will contribute to a better understanding of the development of emerging motor and cognitive skills due to the availability of quantifiable outcome measures. Computer game based rehabilitation platforms have the ability to be easily transferred from a clinical facility based setting to a community or home based setting with the help of telerehabilitation. Telerehabilitation systems can dramatically decrease the cost of providing routine treatments to children. Such systems increase both the range and the number of patients that clinician specialists can attend to in local as well as rural regions. The CUE assessment tool can provide reports related to compliance, effectiveness, and identification of problems, and for the continuum of care for children with CP.

## Conclusion

The high to moderate ICC values and lack of systematic errors in the outcome measures indicate that the CUE assessment tool has the ability to repeatedly record reliable performance measures of many different object manipulation tasks that have a broad range of physical properties and functional demands. Due to the high variation in CUE outcome measures among the participants most MDC values were in the range of 30–60% of the group average. The lack of a significant correlation between CUE performance measures and the QUEST or PDMS-2 indicates that the two assessment tools represent distinct attributes or features of object handling and manipulation skills.
